# Conformational risk factors of brachycephalic obstructive airway syndrome (BOAS) in pugs, French bulldogs, and bulldogs

**DOI:** 10.1371/journal.pone.0181928

**Published:** 2017-08-01

**Authors:** Nai-Chieh Liu, Eileen L. Troconis, Lajos Kalmar, David J. Price, Hattie E. Wright, Vicki J. Adams, David R. Sargan, Jane F. Ladlow

**Affiliations:** 1 Department of Veterinary Medicine, University of Cambridge, Cambridge, Cambridgeshire, United Kingdom; 2 Vet Epi, Mildenhall, Suffolk, United Kingdom; University of Bari, ITALY

## Abstract

Extremely brachycephalic, or short-muzzled, dog breeds such as pugs, French bulldogs, and bulldogs are prone to the conformation-related respiratory disorder—brachycephalic obstructive airway syndrome (BOAS). Affected dogs present with a wide range of clinical signs from snoring and exercise intolerance, to life-threatening events such as syncope. In this study, conformational risk factors for BOAS that could potentially aid in breeding away from BOAS were sought. Six hundred and four pugs, French bulldogs, and bulldogs were included in the study. Soft tape measurements of the head and body were used and the inter-observer reproducibility was evaluated. Breed-specific models were developed to assess the associations between the conformational factors and BOAS status based on functional grading. The models were further validated by means of a BOAS index, which is an objective measurement of respiratory function using whole-body barometric plethysmography. The final models have good predictive power for discriminating BOAS (-) and BOAS (+) phenotypes indicated by the area under the curve values of >80% on the receiver operating curves. When other factors were controlled, stenotic nostrils were associated with BOAS in all three breeds; pugs and bulldogs with higher body condition scores (BCS) had a higher risk of developing BOAS. Among the standardized conformational measurements (i.e. craniofacial ratio (CFR), eye width ratio (EWR), skull index (SI), neck girth ratio (NGR), and neck length ratio (NLR)), for pugs EWR and SI, for French bulldogs NGR and NLR, and for bulldogs SI and NGR showed significant associations with BOAS status. However, the NGR in bulldogs was the only significant predictor that also had satisfactory inter-observer reproducibility. A NGR higher than 0.71 in male bulldogs was predictive of BOAS with approximately 70% sensitivity and specificity. In conclusion, stenotic nostrils, BCS, and NGR were found to be valid, easily applicable predictors for BOAS (+).

## Introduction

Brachycephalic obstructive airway syndrome (BOAS) is a conformation-related respiratory disorder of dog breeds with shortened skulls and muzzles, such as the pug, the French bulldog, the bulldog, and others [1]. Breeding selection for extreme brachycephalia has resulted in deformation of the upper airway tract leading to obstruction, as the soft tissues have not reduced proportionately with the length of the skull [2]. Affected dogs show noisy and laboured breathing with exercise and heat intolerance, often accompanied by sleep disturbed breathing, gastrointestinal disorders such as regurgitation and vomiting, and in the worst cases, cyanosis, collapse and death [3–6]. Although clinical signs of BOAS can, at an individual level, be improved by surgery, severely affected dogs are at a higher anaesthetic risk. Moreover, for dogs that have developed secondary lesions such as collapse of the laryngeal cartilages (Grade II-III laryngeal collapse), the prognosis may be guarded.

BOAS is a serious welfare issue [7, 8]. The average lifespan of brachycephalic breeds is reduced by approximately three years when compared to that of mesaticephalic and dolichocephalic breeds of similar body size [9–12], with much of this difference likely due to BOAS and its syndromic effects. The problems caused by BOAS have been compounded by the increased popularity of the three extreme brachycephalic breeds named above over the last two decades in the UK and elsewhere. Moreover, the drivers that may have caused the increasing numbers of these dogs, such as celebrity endorsement and widespread adoption by advertisers, remain in place. All three breeds are now amongst the top ten breeds in popularity in the UK [13].

In the past century, the skull shape in the extreme brachycephalic breeds has gradually decreased in facial length and increased in skull width proportionally [14], and it has often been suggested that this is associated with an increase in both severity and prevalence of BOAS [15]. In our previous study, we found that approximately 50% of our study dogs in the three extreme brachycephalic breeds were BOAS-affected according to whole-body barometric plethysmography (WBBP), an objective respiratory function test [16]. Others have shown that during a two-year period in the UK, about 20% of pugs have respiratory disease at a level which triggers a veterinary consultation [17]. Given that an estimated 60% of owners do not recognise the clinical signs of BOAS [18, 19], the true prevalence may be much higher. However, it is also true that the high-risk breeds include elderly dogs that have normal respiratory traces and have not suffered from BOAS during their lifespan.

Recently, there has been a growing awareness of BOAS-related welfare issues amongst dog owners and other stakeholders. It has been suggested that reformation of the breed standards of brachycephalic breeds could reduce the prevalence of BOAS in extreme brachycephalic breeds. Several studies have identified and quantified the causal anatomical lesions along the upper airway using advanced diagnostic images such as computed tomography (CT) and endoscopy. Quantitative measurements were described in order to look for potential biomarkers for BOAS. These include the length and thickness of the soft palate [20], the tracheal diameter [21], nasopharyngeal dimension [22], glottis dimension [23], and mucosal contact points of the nasal turbinates [24]. However, these measurements required sedation or general anaesthesia, which is impractical for screening the pet population—particularly in dogs that have no clinical signs of BOAS. Alternatively, soft tape measurements have been proposed to quantify morphology in dogs [25] and were further applied to study BOAS [26]. The Packer *et al*. (2015) study reported that craniofacial ratio (CFR) and neck girth are conformation-related risk factors for BOAS when comparing data across breeds. These measurements are non-invasive and easily accessible. The practical implications of these measurements in specific breeds is still uncertain, and their reproducibility is also uncertain. Hence there is an urgent need for anatomical or conformational markers that can allow breeders of these short-faced dogs to select away from BOAS.

The objective of this study was to identify breed specific external conformational characteristics that are associated with BOAS. Soft tape measurements were used to quantify conformational features. Inter-observer reproducibility of the measurements was examined to see whether these measurements could form a secure basis for breeder decisions. The conformational measurements and other potential factors such as body condition score (BCS) were then compared with BOAS Functional Grade (i.e. clinical assessment of respiratory signs before and after an exercise tolerance test) and the associations were further validated using the BOAS index (i.e. objective score of respiratory obstruction measured from WBBP parameters) developed using breed-specific computational models (16).

## Materials & methods

### Animals

Six hundreds and four dogs (189 pugs, 214 French bulldogs, 201 bulldogs) were included in this study. The subjects were either: referred for BOAS consultation at the Queen’s Veterinary School Hospital (QVSH), University of Cambridge; were pet dogs volunteered by UK owners and breeders; were show dogs exhibited at regional dog shows between September 2013 and September 2016. Dogs were excluded if: they were aged <1 year of age as they were considered immature in head and body dimension; they had had previous upper airway surgery; they had history/clinical findings of lower airway disease. Dogs that were on medications that may change respiration (e.g. prednisolone and anti-inflammatory drugs) were also excluded from the study. A detailed history of each dog was taken from owners including type, severity, frequency and circumstances of occurrence of respiratory signs. Work was performed under informed ethical consents CR62 and CR63 from the Department of Veterinary Medicine, University of Cambridge.

### Respiratory grading and BOAS Index

All participating dogs were graded for functional severity of BOAS using a previously established four-point functional grading system based on clinical evaluation before and after an exercise tolerance test [16, 19]. BOAS functional *Grade 0* dogs (asymptomatic, BOAS free) and *Grade I* dogs (mild BOAS, the dog shows mild respiratory noise but exercise tolerance is unaffected) were considered not to have clinically significant BOAS, namely **‘BOAS (-)’** in this study; *Grade II* dogs (moderate BOAS, the dog requires medical attention such as weight control and/or surgical intervention) and *Grade III* dogs (severe BOAS, the dog requires immediate surgical intervention) were considered clinically affected, namely **‘BOAS (+)’** in this study.

The **BOAS index** (a numeric scale of 0–100%, with 0% being entirely asymptomatic and 100% being the most severe status of BOAS), derived from previously established breed-specific computational models [16] using quadratic discriminant analysis, computed from WBBP waveforms of some participating dogs, was used to validate the risk predictive models (for details see the section ‘statistical analysis’). Not all participants had available WBBP data due to an insufficient testing period, a distracting testing environment, and/or test intolerance of the animal. The detailed protocols of the WBBP test were reported previously [16].

### Conformational classifications and measurements

**Fig 1:**
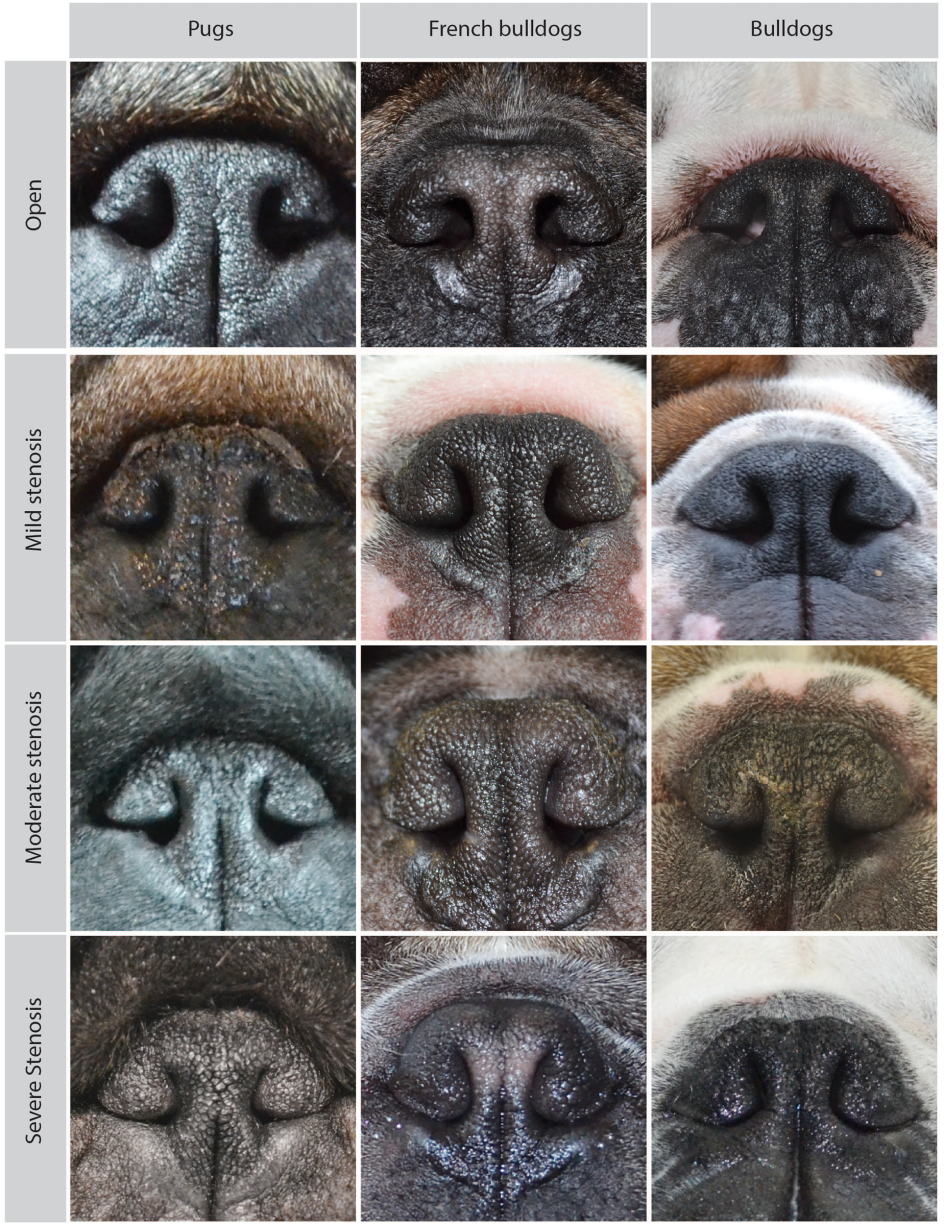
Examples of different degrees of nostril stenosis in pugs, French bulldogs, and bulldogs. The following descriptions were adapted from a previously published figure by the authors (Fig 1 in Liu *et al*. 2016): “Open nostrils: nostrils are wide open; mildly stenotic nostrils: slightly narrowed nostrils where the lateral nostril wall does not touch the medial nostril wall. Immediately after the exercise tolerance test (ETT), the nostril wings should move dorsolaterally to open on inspiration; moderately stenotic nostrils: the lateral nostril wall touches the medial nostril wall at the dorsal part of the nostrils and the nostrils are only open at the bottom. Immediately after the ETT, the nostril wings are not able to move dorsolaterally and there may be nasal flaring (ie, muscle contraction around the nose trying to enlarge the nostrils; severely stenotic nostrils: nostrils are almost closed. The dog may switch to oral breathing from nasal breathing with stress or very gentle exercise such as playing.” (Liu *et al*. 2016).

For all dogs, a body weight measurement, and a standard assessment of the body fat, the **body condition score (BCS)** on a 1–9 point scale [27], was performed. The degree of nostril stenosis was examined and classified using a previously established grading system [16]. “Open or mild” stenosis was considered acceptable for these breeds, and “moderate or severe” stenosis was defined as **‘stenotic nostrils’** in this study [28]. Examples of the nostril grading for each breed are shown in Fig 1.

**Fig 2:**
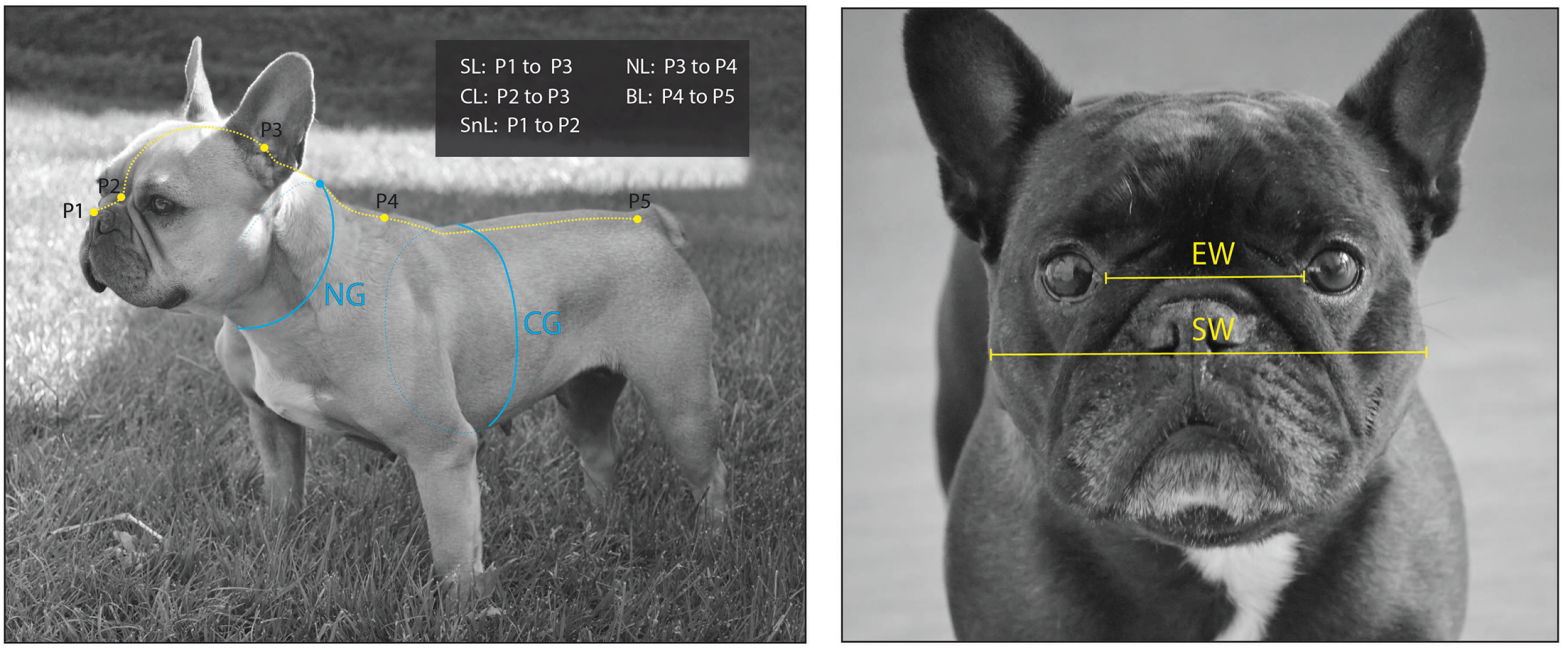
Demonstration of the soft tape measurements. Nine measurements were made with a firmly held soft tape measure with the dog standing and at rest: Skull length (SL), cranial length (CL), snout length (SnL), neck length (NL), body length (BL), eye width (EW), and skull width (SW). The detailed definitions of the measurements are shown in Table 1. The photos of the dogs were taken from two of the study dogs by the authors.

Nine measurements of the skull and body of each dog were taken using a standard one-meter soft tape measure (millimeter scales): **skull length (SL), cranial length (CL), snout length (SnL), skull width (SW), eye width (EW), neck length (NL), neck girth (NG), chest girth (CG), and body length (BL)**. The definitions of each measurement are listed in Table 1, while Fig 2 illustrates the method of measurement. Some of these measurements were described previously [25]. SL and SW were used to estimate the skull index, which was used as an indicator of different skull shapes: brachycephalic, mesaticephalic, or dolichocephalic [29].

**Table 1:** Descriptions of the soft tape measurements.

	Descriptions
**Cranial length (CL)**	The distance along the surface of the head at the skull midline from the external occipital protuberance to the point between the medial canthus of the left eye and the medial canthus of the right eye.
**Snout length (SnL)**	The distance along the surface of the head at the skull midline from the stop to the rostral end of the nasal planum.
**Skull length (SL)** *	The distance along the surface of the head at the skull midline from the external occipital protuberance to the rostral end of the nasal planum.
**Skull width (SW)**	The linear distance (widest distance) between the left external zygomatic arch and the right external zygomatic arch.
**Eye width (EW)**	The linear distance between the medial canthus of the left eye and the medial canthus of the right eye.
**Neck length (NL)**	The distance along the dorsal body midline from the external occipital protuberance to the point between the cranial angle of the left scapula and the cranial angle of the right scapula.
**Neck girth (NG)**	The circumference of the neck at the median distance between the external occipital protuberance and the point between the cranial angle of the left scapula and the cranial angle of the right scapula.
**Chest girth (CG)**	The circumference of the deepest part of the thoracic cavity.
**Body length (BL)**	The distance along the dorsal body midline from the the point between the cranial angle of the left scapula and the cranial angle of the right scapula to the root of the tail.

* Skull length measurement in this study was the sum of CL measurement and SnL measurement

### Inter-observer reproducibility of the conformational soft tape measurements

Inter-observer reproducibility of each measurement was tested for each breed separately due to their different facial and body conformations. Sixty dogs (20 dogs for each breed) were included in the inter-observer reproducibility test. The bulldogs were from a single dog show, while the pugs and French bulldogs were from volunteered dogs and referral dogs measured at the QVSH. For each dog, soft tape measurements were repeated by two investigators who had been trained to perform the measurements. We randomly combine the two observers from a group of four investigators to avoid the inclusion of systematic errors and better model a potential pair of breeders.

### Statistical analysis

All Statistical analyses were performed using R (version 3.3.0 for Mac, https://www.r-project.org). Significance level was set at 0.05 in all tests, unless otherwise indicated.

Comparisons of numeric variables between breeds were performed using analysis of variance (ANOVA) with a post-hoc test for pairwise comparison. Categorical variables were compared using Chi-square tests, followed by multiple pairwise comparisons. Bonferroni corrections were used in each case.

Due to facial and body differences between breeds, all of the following analyses were completed within each breed.

#### (1) Inter-observer reproducibility of soft tape measurements

To evaluate the inter-observer agreement, the intra-class correlation coefficient (ICC) [30] for each measurement of each breed was calculated using R’s package “irr” [31]. Two-way models were used and the reproducibility between the observers were estimated. The test of agreement was chosen instead of the test consistency (raw R function code can be found in https://rdrr.io/cran/irr/src/R/icc.R). This decision was made as in our case the mean values were of interest when assessing the difference. At least 18 subjects were required to assess ICC. This sample size was calculated using R’s package “ICC.Sample.Size” and function “calculateIccSampleSize” with two raters, at 5% significance, 80% power and with preliminary estimate of the CFR’s ICC at 0.6 across breeds. To evaluate the variation in measurement difference between the two observers, the estimated mean of random measurement error (i.e. the ratio of measurement difference between the two observations to the mean of the observations, eME) of each measurement was calculated for each breed. The inter-observer agreement of the measurements was judged based on the ICC value with the criteria as follows [32]:

**Excellent inter-observer agreement**: ICC greater than 0.9
**Good inter-observer agreement**: ICC between 0.75 and 0.9
**Moderate inter-observer agreement**: ICC between 0.5 and 0.75
**Poor inter-observer agreement**: ICC less than 0.5

#### (2) Predictive models of conformational risk factors for BOAS

Principle component analysis was used initially to group soft tape measures as a means of reducing the number of variables, but this did not improve the fit significantly. For further modelling, five ratios of the measurements were calculated that partially compensate for body size within breeds:

**Craniofacial ratio (CFR)**: snout length (SnL) / cranial length (CL)
**Eye width ratio (EWR)**: eye width (EW) / skull width (SW)
**Skull index (SI)**: skull width (SW)/ skull length (SL)
**Neck girth ratio (NGR)**: neck girth (NG) / chest girth (CG)
**Neck length ratio (NLR)**: neck length (NL) / body length (BL)

Eleven predictive variables of interest were included in breed-specific multivariate logistic regression models initially, namely: age (months; numeric variable), gender (male/female, binary variable), neuter status (yes/no; binary variable), body weight (kilograms; numeric variable), BCS (scaled 1–9; numeric variable), stenotic nostrils (yes/no; binary), CFR (in proportion; numeric variable), EWR (in proportion; numeric variable), SI (in proportion; numeric variable), NGR (in proportion; numeric variable), and NLR (in proportion; numeric variable) where the BOAS status (BOAS (-) and BOAS (+); binary variable) was the outcome variable. Backwards stepwise model-selection based on Akaike’s Information Criterion (AIC) [33] was used to obtain the best-fit model. Gender was a variable of interest; even if it did not improve the final model significantly, it was retained in the model. The estimated probability of BOAS (+) was computed for each dog from the models. Receiver operating characteristic (ROC) curves were used to evaluate the predictive performance of the breed-specific models based on the area under the ROC curve (AUC).

Conformational predictors that were retained in the final model and had satisfactory inter-observer agreement (good or excellent ICC) were further investigated to establish a threshold for predicting BOAS that can easily be implemented by breeders. Threshold values for the conformational measurements were calculated for male and female separately (due to the significant difference between gender) based on maximizing the sum of sensitivity and specificity from ROC curves.

#### (3) Validation of the conformational risk factors

A validation test was performed to test whether the predictive variables that were retained in the logistic regression models (see previous section) predict BOAS index. BOAS indices were available for only about half of the dogs (115/189 pugs, 100/214 French bulldogs, and 79/201 bulldogs). The BOAS (+) prevalence was compared between the total study population (i.e. all study dogs) and the validation population (i.e. the ones that had a BOAS index available) for each breed using a Chi-squared test. The variables were fitted into a multivariate linear regression where BOAS index (%) was the outcome variable. The adjusted R-squared was calculated for each breed-specific model.

## Results

### Subjects

Details of the signalment are shown in Table 2. Pugs had significantly higher BCS (median BCS = 7) than French bulldogs (median BCS = 5, p<0.0001) and bulldogs (median, BCS = 6, p<0.0001). There was no significant difference in gender distribution between breeds (p = 0.736). The bulldog group had a significantly lower proportion of dogs that were neutered when compared to the pug (χ^2^ = 40.41, p<0.0001) and the French bulldog groups (χ^2^ = 32.12, p<0.0001). The distribution of the degree of nostril stenosis was significantly different between the three breeds (χ^2^ = 43.28, p<0.0001). 75.4% of French bulldogs had moderately to severely stenotic nostrils, while prevalence amongst pugs (65.3%) and bulldogs (44.2%) was lower. The proportion of BOAS (+) pugs (64.6%) was higher than the French bulldog (58.9%) and the bulldog (51.2%) groups. However, the distribution of the BOAS functional grades did not differ significantly between breeds.

**Table 2:** Signalment and the proportions of the subjects with stenotic nostrils and different functional grades.

	Pug	French bulldog	Bulldog
**Number**	189	214	201
**Study population**	Clinical: 14.8%	Clinical: 17.3%	Clinical: 4.5%
	Volunteered: 85.2%	Volunteered: 82.7%	Volunteered: 95.5%
**Age** (months, median with range)	36 (12–147)	28 (12–126)	26 (12–178)
**Gender**	Male: 40.7%	Male: 44.4%	Male: 43.8%
	Female: 59.3%	Females: 55.6%	Female: 56.2%
**Neuter status**	Intact: 64.6%	Intact: 68.7%	Intact: 91.5%
	Neutered: 35.4%	Neutered: 31.3%	Neutered: 8.5%
**Body weight** (kg, mean ± SD)	8.53 ± 1.47	12.12 ± 2.05	25.47 ± 3.11
**BCS** (median with range; obesity *)	7 (4–9); 60.8%	5 (3–9); 8.4%	6 (4–8); 35.3%
**Degree of nostril stenosis**	Open: 9.5%	Open: 10.8%	Open: 26.9%
	Mild: 21.2%	Mild: 13.6%	Mild: 28.4%
	Moderate: 38.1%	Moderate: 29.0%	Moderate: 34.3%
	Severe: 19.6%	Severe: 45.33%	Severe: 9.5%
	NA: 11.6%	NA: 1.4%	NA: 1.0%
**BOAS Functional Grade**	Grade 0: 4.8%	Grade 0: 10.7%	Grade 0: 10.9%
	Grade I: 30.7%	Grade I: 30.4%	Grade I: 37.8%
	Grade II: 44.9%	Grade II: 43.5%	Grade II: 38.8%
	Grade III: 19.6%	Grade III: 15.4%	Grade III: 12.4%

SD, standard deviation; BCS, body condition score; BOAS, brachycephalic obstructive airway syndrome; NA, data not available.

* Obesity was defined as BCS ≥ 7 on a 9-point scale.

As all analyses were carried out for each individual breed, results are presented separately for each breed.

### Pugs

#### (1) Inter-observer reproducibility of soft tape measurements

**Fig 3:**
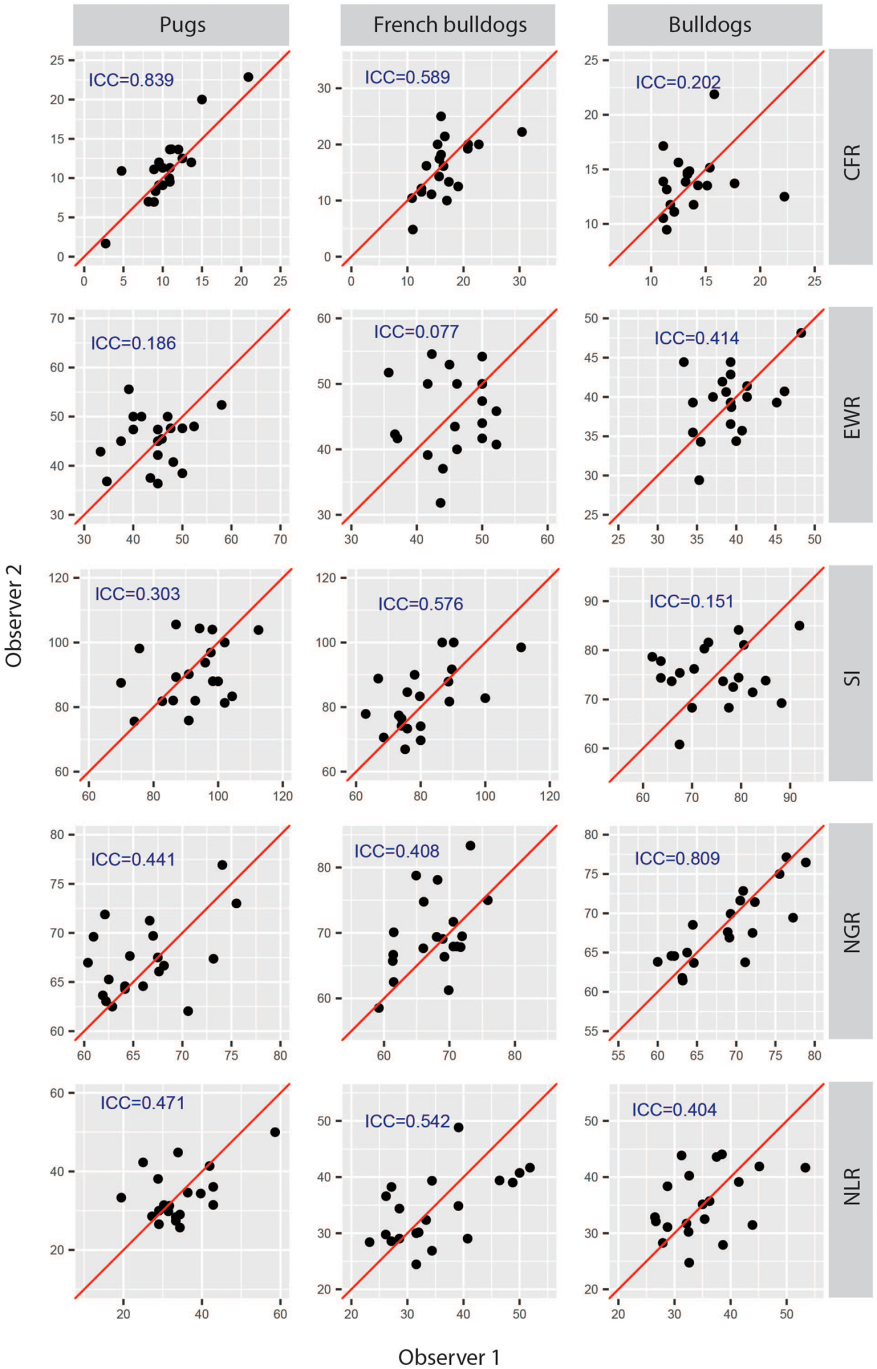
Scatter plots show the inter-observer reproducibility of the conformational soft tape measures. The diagonal red line indicates perfect agreement. ICC, intra-class correlation coefficient; CFR, craniofacial ratio; EWR, eye width ratio; SI, skull index; NGR, neck girth ratio; NLR, neck length ratio.

The inter-observer variations in soft tape measurements and the results of inter-observer reproducibility are shown in S1 and S2 Tables (raw data can be found in S1 Data). Overall, most of the measurements and their ratios had poor inter-observer agreement (ICC<0.5) between the two observers, except for SnL (ICC = 0.83; 95%CI: 0.62 to 0.93) and CG (ICC = 0.83; 95%CI: 0.62 to 0.93) (Fig 3). Among the ratios, only CFR had good inter-observer agreement with the estimated ICC = 0.84 (95%CI: 0.64 to 0.93). However, it is worth noting that although CFR had good measurement agreement, its eME was high at 18.9 ± 17.7%. CFR was the ratio of SnL to CL, where CL had poor inter-observer agreement (ICC = 0.4, 95%CI: -0.05 to 0.71), while SnL had relatively good inter-observer agreement but with high eME (likely due to the relatively large difference between the two measurements to its relatively small absolute value: mean = 1.16cm). Consequently, the inter-observer agreement of CFR measurement was questioned.

#### (2) Soft tape measurement of conformation in relation to BOAS

The summary of each soft tape measurement with BOAS functional grade can be seen in S3 Table. Substantial overlaps are present in all ratios among different functional grades (S1 Fig). Among the direct measurements, the BOAS (+) dogs had significantly greater EW than the BOAS (-) pugs (p = 0.009). There is no significant difference in any ratios between BOAS (-) and BOAS (+).

#### (3) Full model of conformation, nostril stenosis and BCS predicting BOAS

**Fig 4:**
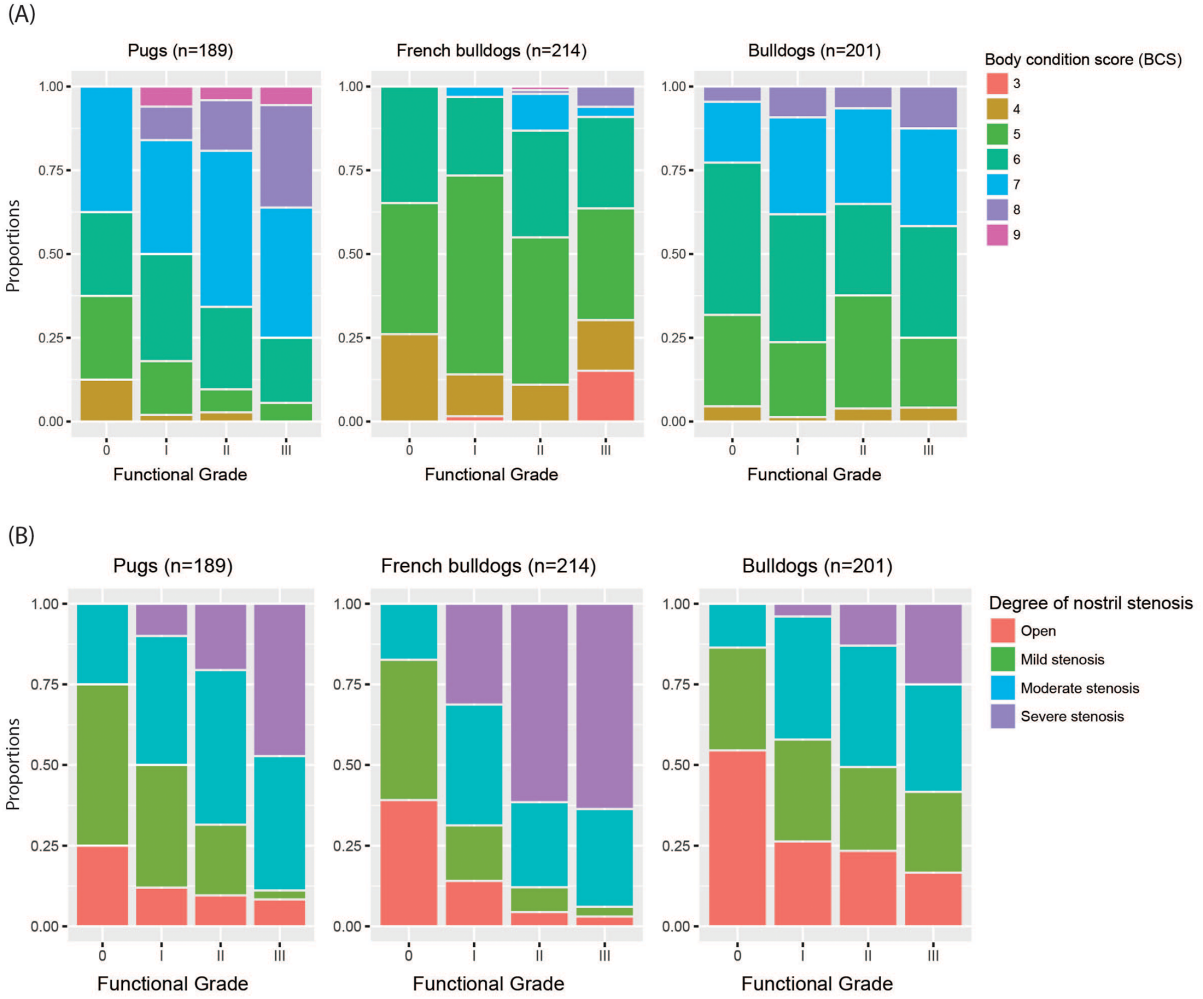
Stacked bar charts demonstrate the relationship between BOAS functional grade and body condition score (BCS), and the degree of nostril stenosis. The y-axis shows the percentage of dogs of that functional grade showing each degree of BCS or nostril stenosis. Note the clear trends of an increased proportion of obese dogs and dogs with moderately or severely stenotic nostrils with an increase in functional grade. In French bulldogs, there were over 10% of Grade III dogs that had BCS of 3. The underweight condition was attributed to BOAS-related frequent regurgitation.

The results of the multivariate logistic regression models are shown in Table 3 (raw data can be found in S2 Data). The final model for pugs contained seven variables that accounts for 48% of the total variation in predicting BOAS (+). Female pugs had 5.35 (95%CI: 2.2 to 13.9) times greater odds than males of being BOAS (+), after adjusting for the other factors. Pugs with moderately/severely stenotic nostrils had 4.58 (95%CI: 2.11 to 10.4) times greater odds of being BOAS (+) than those with open/mildly stenotic nostrils. Fig 4 shows a clear trend that the higher the functional grade the higher the proportion of dogs with moderate/severe nostril stenosis.

**Table 3:** The breed-specific models predicting the probability of having brachycephalic obstructive airway syndrome (BOAS).

	B (SE)	z value	Odd ratio (95%CI)	p value
***Model (Pug)*: *pseudo-R***^***2***^ **=** ***0*.*48***				
(Intercept)	-12.959 (3.749)	-	-	-
Gender	-1.676 (0.468)	-3.584	0.187 (0.072 to 0.454)	<0.001 ***
BCS	0.363 (0.196)	1.849	1.437 (0.983 to 2.131)	0.064
Stenotic nostrils	1.521 (0.405)	3.759	4.579 (2.110 to 10.396)	<0.001 ***
Body weight (kg)	0.460 (0.176)	2.611	1.584 (1.135 to 2.272)	0.009 **
EWR	0.081 (0.038)	2.147	1.084 (1.009 to 1.171)	0.032 *
SI	0.068 (0.021)	3.238	1.070 (1.028 to 1.117)	0.001 **
NLR	-0.070 (0.037)	-1.869	0.933 (0.866 to 1.003)	0.062
***Model (French bulldog)*: *pseudo-R***^***2***^ **=** ***0*.*37***				
(Intercept)	-7.563 (2.70)	-	-	-
Age (month)	0.011 (0.007)	1.547	1.011 (0.997 to 1.026)	0.122
Gender	0.757 (0.340)	2.227	2.132 (1.102 to 4.198)	0.026 *
BCS	0.256 (0.177)	1.447	1.292 (0.919 to 1.846)	0.148
Stenotic nostrils	1.731 (0.397)	4.360	5.645 (2.649 to 12.676)	<0.0001 ***
CFR	-0.065 (0.046)	-1.429	0.937 (0.855 to 1.023)	0.153
NGR	0.115 (0.035)	3.265	1.122 (1.049 to 1.206)	0.001 **
NLR	-0.071 (0.029)	-2.431	0.932 (0.879 to 0.986)	0.015 *
***Model (Bulldog)*: *pseudo-R***^***2***^ **=** ***0*.*37***				
(Intercept)	-24.119 (4.224)	-	-	-
Gender	0.120 (0.386)	0.311	1.127 (0.524 to 2.390)	0.756
Neuter status	2.091 (0.727)	2.876	8.093 (2.142 to 38.94)	0.004 **
BCS	0.441 (0.193)	2.287	1.555 (1.073 to 2.295)	0.022 *
Stenotic nostrils	0.463 (0.344)	1.349	1.590 (0.811 to 3.132)	0.177
SI	0.045 (0.019)	2.403	1.046 (1.009 to 1.086)	0.016 *
NGR	0.255 (0.050)	5.075	1.290 (1.175 to 1.431)	<0.0001 ***

Multivariate logistic regression was used with BOAS (+) and BOAS (-) as the binary outcome variable.

**B**, regression coefficient; **SE**, standard error; **CI**, confidence interval; **BCS**, body condition score; **EWR**, eye width ratio = eye width /skull width; **SI**, skull index = skull width / skull length; **NLR**, neck length ratio = neck length / body length; **CFR**, craniofacial ratio = snout length / cranial length; **NGR**, neck girth ratio = neck girth / chest girth.

When including the ratios (EWR, SI, NLR, CFR, NGR), in the above models, the values were converted from a proportion/ratio to a percentage (i.e., the ratio times 100) so that the coefficients were easier to read.

The **binary outcome variable** used in the multivariate logistic regression was based on BOAS functional grades: 0 = function grade 0/I; 1 = functional grade II/III

For the binary variable the coding was defined as follows:
**Gender** (0 = female; 1 = male) **Stenotic nostrils** (0 = open or mildly stenotic nostrils; 1 = moderately or severely stenotic nostrils) **Neuter status** (0 = intact; 1 = neutered)

* The significance level was set at p<0.05;

** p<0.01,

*** p<0.001.

With regards to the obesity-related variables, BCS was retained in the final model although it was not significant (p = 0.064). Nevertheless, the estimated odds ratio was 1.44 with the upper limit of its 95%CI at 2.13, thus the impact of BCS on BOAS could still be clinically significant. There was a clear trend that the higher the functional grade, the higher the proportion of high-BCS (Fig 4).

**Fig 5:**
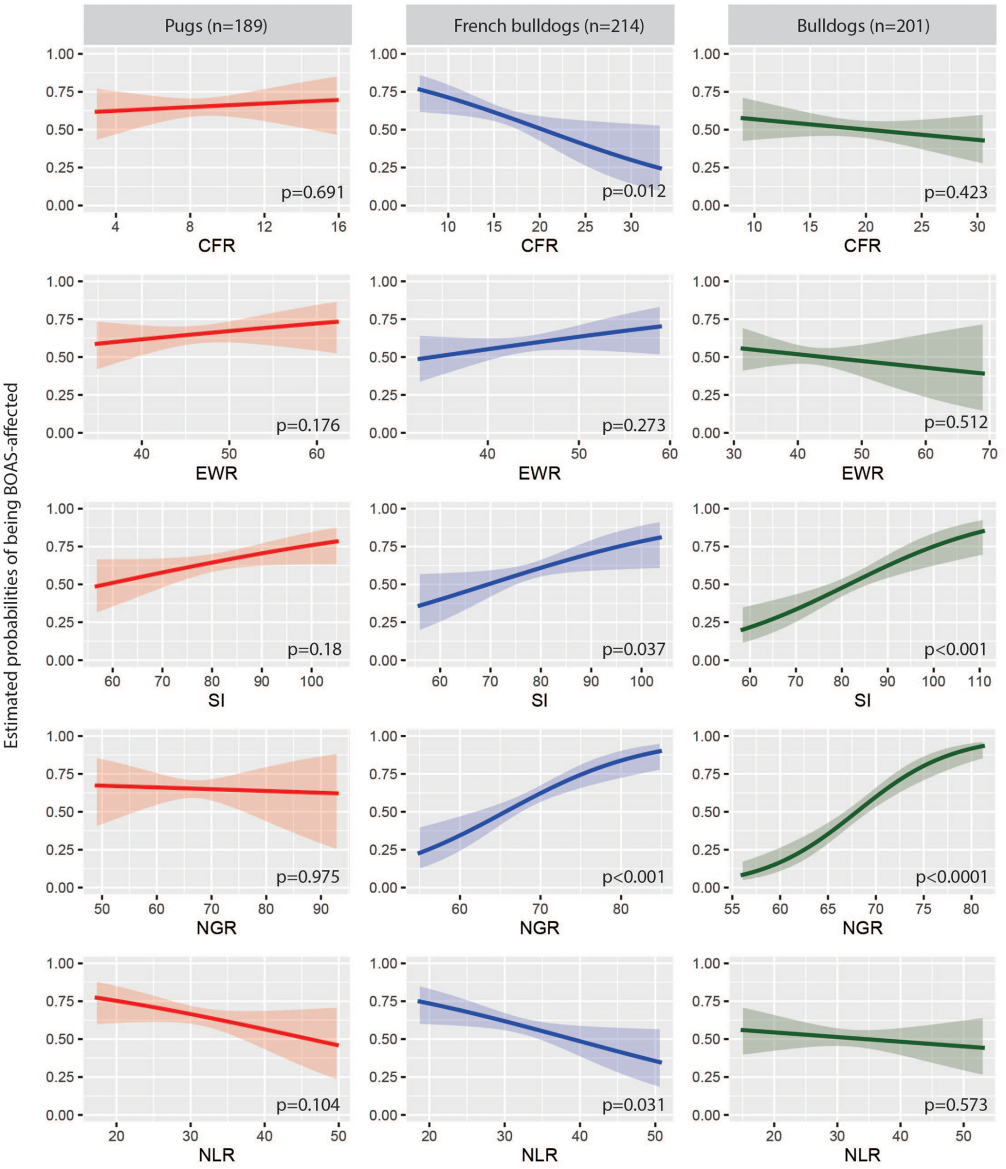
Univariate logistic regression plots demonstrate the trend of the five conformational ratios against the estimated BOAS probability. The x-axis is the numeric data of each ratio in percentage; the y-axis is the estimated probability of BOAS. CFR, craniofacial ratio; EWR, eye width ratio; SI, skull index; NGR, neck girth ratio; NLR, neck length ratio.

Conformational factors EWR (p = 0.032) and SI (p = 0.001) were significantly associated with BOAS after adjusting for other factors. There was a tendency for dogs with higher EWR and SI to be more likely BOAS (+) (Table 3). However, we note that both of these measurements showed poor inter-observer agreement that question their validity as predictors (S1 and S2 Tables). Fig 5 gives an indication of the effect of each ratio on the probability of BOAS; depicting the univariate logistic curve fit to each ratio individually.

**Fig 6:**
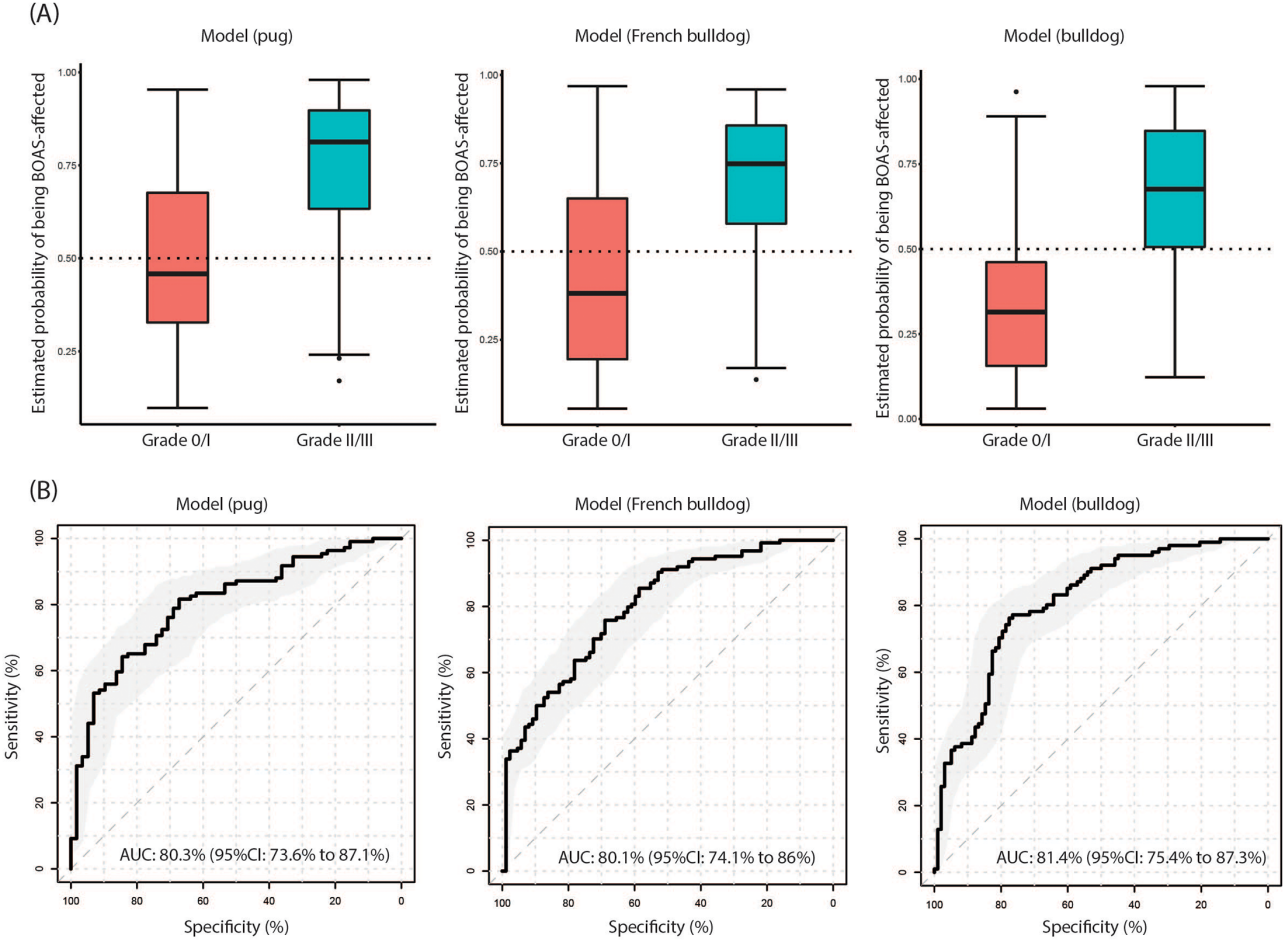
The predictive performance of the breed specific models. (A) boxplots show the distributions of the estimated probability of being BOAS-affected. The dotted line at 50% of the probability represents the raw predictive cut-off. For pugs and French bulldogs, the cut-off values can be adjusted to improve the specificity of the models; (B) receiver operating characteristic (ROC) curves show the predictive performance of the breed specific models. Area under the curve (AUC) was computed with its 95% confidence interval in the bracket.

Fig 6 shows the predictive performance of the final model. The classification was based on a cut-off value of 0.5 (Fig 6A; that is, a predicted probability of disease > 0.5 meant the dog was classified as positive, otherwise, negative). This could be adjusted depending on the users’ preference for sensitivity or specificity. The ROC curve had an AUC of 80% (95%CI: 74% to 87%); considered as good accuracy in classifying BOAS (+) and BOAS (-) dogs.

#### (4) Validation of the model using BOAS index

The BOAS (+) prevalence (functional grade II/III) was not significantly different between the total study pugs (i.e. all study pugs) and the validation population (i.e. the ones that had BOAS index available) (χ^2^ = 0.183, p = 0.669). The results of the multivariate linear regression model can be found in Table 4 (raw data can be found in S3 Data). The adjusted R-squared value of the multivariate linear regression model was 0.2, indicating the proportion of variability in BOAS index that can be explained by the models. The predictive factors that were significant in the validation model were similar to the full model (Table 3), where female pugs (p = 0.03), pugs with moderate/severe stenotic nares (p = 0.007), and higher SI (p = 0.044) are more likely to have higher BOAS index. Interestingly, while body weight was not significant in the validation model (p = 0.36), BCS was significant (p = 0.007) in predicting BOAS index: with one unit increase in BCS corresponding to an increased BOAS index of 6.4%, on average (all else constant).

**Table 4:** Validation of the predictive models using BOAS index.

	B (SE)	95%CI	t value	p value
**Validation_Model (Pug): R**^**2**^ **=** **0.20**				
(Intercept)	-27.968 (37.925)	-	-	-
Gender	-11.128 (5.046)	-21.137 to -1.119	-2.205	0.030 *
BCS	6.417 (2.203)	2.047 to 10.786	2.913	0.004 **
Stenotic nostrils	12.921 (4.681)	3.637 to 22.205	2.760	0.007 **
Body weight (kg)	1.717 (1.867)	-1.987 to 5.421	0.919	0.360
EWR	0.249 (0.423)	-0.591 to 1.088	0.588	0.558
SI	0.442 (0.217)	0.011 to 0.873	2.035	0.044 *
NLR	-0.623 (0.411)	-1.437 to 0.192	-1.517	0.132
**Validation_Model (French bulldog): R**^**2**^ **=** **0.20**				
(Intercept)	-37.271 (39.269)	-	-	-
Age (month)	0.071 (0.10)	-0.128 to 0.271	0.710	0.479
Gender	-5.460 (5.395)	-16.175 to 5.255	-1.012	0.314
BCS	0.524 (2.650)	-4.740 to 5.788	0.198	0.844
Stenotic nostrils	16.490 (5.775)	5.020 to 27.960	2.855	0.005 **
CFR	-0.633 (0.666)	-1.955 to 0.690	-0.950	0.345
NGR	1.674 (0.530)	0.622 to 2.726	3.160	0.002 **
NLR	-0.848 (0.433)	-1.707 to 0.012	-1.959	0.053
**Validation_Model (Bulldog): R**^**2**^ **=** **0.26**				
(Intercept)	-180.142 (53.314)	-	-	-
Gender	-4.725 (6.765)	-18.211 to 8.760	-0.699	0.487
Neuter status	23.737 (11.561)	0.690 to 46.784	2.053	0.044 *
BCS	4.427 (2.849)	-1.251 to 10.105	1.554	0.125
Stenotic nostrils	16.457 (5.423)	5.646 to 27.267	3.034	0.003 **
SI	0.402 (0.260)	-0.115 to 0.919	1.549	0.126
NGR	2.339 (0.698)	0.948 to 3.731	3.351	0.001 **

Multivariate regression was used with BOAS index as the outcome variable.

To validate the predictive models (Table 3) that the outcome variable was based on a subjective clinical assessment (functional grading system), the objective respiratory function severity score, BOAS index (a numeric score computed from flow waveforms obtained from whole-body barometric plethysmography), was further used on a smaller study population (pug = 115; FB = 100; bulldog = 79). Variables that were included in the predictive models in Table 3 were input into the multivariate regression models.

**BOAS**, brachycephalic obstructive airway syndrome; **B**, regression coefficient; **SE**, standard error; **CI**, confidence interval; **BCS**, body condition score; EWR, eye width ratio = eye width/ skull width; **SI**, skull index = skull width / skull length; **NLR**, neck length ratio = neck length / body length; **CFR**, craniofacial ratio; **NGR**, neck girth ratio = neck girth / chest girth;

When including the ratios (EWR, SI, NLR, CFR, NGR), in the above models, the values were converted from a proportion/ratio to a percentage (i.e., the ratio times 100) so that the coefficients were easier to read.

For the binary variable the coding was defined as follows:
**Gender** (0 = female; 1 = male) **Stenotic nostrils** (0 = open or mildly stenotic nostrils; 1 = moderately or severely stenotic nostrils) **Neuter status** (0 = intact; 1 = neutered)

* The significance level was set at p<0.05

** p<0.01

### French bulldogs

#### (1) Inter-observer reproducibility of soft tape measurements

The inter-observer variations in soft tape measurements are shown in S1 and S2 Tables (raw data can be found in S1 data). Overall, most of the measurements and their ratios had poor to moderate agreement between the two observers. NG (ICC = 0.89, 95%CI: 0.74 to 0.95) and CG (ICC = 0.91, 95%CI: 0.78 to 0.96) measurements had good to excellent inter-observer agreement, but their ratio, NGR, had poor inter-observer agreement (ICC = 0.41, 95%CI: 0 to 0.71), which is likely contributed by the combinations of variances from NG and CG (Fig 3).

#### (2) Soft tape measurement of conformation in relation to BOAS

The summary of each soft tape measurement with BOAS functional grade can be seen in S3 Table. Overall, the BOAS (+) dogs had significantly shorter SnL (p = 0.035), wider SW (p<0.001), wider EW (p<0.001), larger NG (p<0.0001), and longer BL (p = 0.047) than BOAS (-) dogs. In terms of ratios, the BOAS (+) French bulldog had significantly lower CFR (p = 0.012) and NLR (p = 0.034), and higher values of SI (p = 0.042) and NGR (p<0.0001). However, the distribution of ratios was similar to the pugs in that there are substantial overlaps in ratios among different functional grades (S1 Fig). Nonetheless, it is worth noting that the four French bulldogs that had CFR > 0.3 were all Grade 0.

#### (3) Full model of conformation, nostril stenosis and BCS predicting BOAS

The results of the multivariate logistic regression model are shown in Table 3 (Raw data can be found in S2 Data). The final model for French bulldogs contained seven variables that accounted for 37% of the total variation in predicting BOAS (+). Male French bulldogs had 2.13 (95%CI: 1.1 to 4.2) times greater odds of being BOAS (+), than females. French bulldogs that had moderately/severely stenotic nostrils had 5.65 (95%CI: 2.65 to 12.68) times greater odds of being BOAS (+) than those with open/mildly stenotic nostrils. Fig 4 shows a clear trend that the higher the functional grade the higher the proportion of dogs with moderate/severe nostril stenosis.

Fig 5 gives an indication of the effect of each conformational ratio on the probability of BOAS; depicting the univariate logistic curve fit to each ratio individually. NGR was significantly associated with BOAS in French bulldogs, with odds ratios of 1.12 (95%CI: 1.05 to 1.21) for an increase of 0.01 in NGR, respectively. NLR was significantly associated with BOAS, with an odds ratio of 1.07 (95% CI: 1.01 to 1.14) for a decrease of 0.01 in NLR. In addition, there was a tendency for a reduction in CFR to be associated with BOAS, although this was not significant (p = 0.153): the estimated odds ratio was 1.07 (95% CI: 0.98 to 1.17) for a decrease of 0.01 in CFR. BCS was retained in the best-fit model, however, it was not significant (p = 0.148). There was no clear relationship between BCS and BOAS functional grade (Fig 4). Note that, in French bulldogs, 15% of Grade III dogs had BCS of 3, considered as underweight, which accounts for the majority of underweight French bulldogs among the population.

Fig 6 shows the predictive performance of the final model. The classification was based on a cut-off value of 0.5 as before (Fig 6A). The model had AUC of ROC at 80% (95%CI: 74% to 86%), considered as good accuracy in classifying BOAS (+) and BOAS (-) dogs.

#### (4) Validation of the model using BOAS index

The BOAS (+) prevalence (functional grade II/III) was not significantly different between the total study French bulldogs (i.e. all study French bulldogs) and the French bulldogs in the validation population (i.e. the ones that had BOAS index available) (χ^2^ = 0.022, p = 0.883). The results of the multivariate linear regression model can be found in Table 4 (Raw data can be found in S3 Data). The adjusted R-squared value of the multivariate linear regression model was 0.2, indicating the proportion of variability in BOAS index that can be explained by the models. The degree of stenotic nostrils was a significant predictor (p = 0.005). Dogs with moderately/severely stenotic nostrils (compared to open/mild) had a mean increase in BOAS index of 16%. The NGR was significantly associated with BOAS index (p = 0.002); a 0.01 increase in NGR increases BOAS index by 1.67% on average.

### Bulldogs

#### (1) Inter-observer reproducibility of soft tape measurements

The inter-observer variations in soft tape measurements are shown in S1 and S2 Tables (raw data can be found in S1 Data). Overall, most of the direct measurements and their ratios had poor to moderate agreement between the two observers except for CG. Among other ratios, only NGR had good inter-observer agreement with the estimated ICC = 0.81 (95%CI: 0.58 to 0.92) (Fig 3) and its eME was only a mere 3.7% (S1 Table).

#### (2) Soft tape measurement of conformation in relation to BOAS

The summary of each soft tape measurement with BOAS functional grade can be seen in S3 Table. SW (p<0.001), EW (p = 0.014), and NG (p<0.0001) were significantly greater in BOAS (+) bulldogs, who also had significantly higher SI (p<0.001) and NGR (p<0.0001). Similar to the other two breeds, there are substantial overlaps in all ratios among different functional grades (S1 Fig).

#### (3) Full model of conformation, nostril stenosis and BCS predicting BOAS

The results of the multivariate logistic regression model are shown in Table 3 (Raw data can be found in S2 Data). The final model for bulldogs contained six variables that accounted for 37% of the total variation in predicting BOAS (+). Bulldogs that were neutered had 8.1 times greater the odds of being BOAS (+). However, the 95% CI was wide (2.14 to 38.94), which is likely due to the higher proportion of dogs that were not neutered (91.5%). With regards to the obesity-related variables, BCS was significant in the model (p = 0.022) with odds ratio at 1.56 (95%CI: 1.07 to 2.3). Fig 4 demonstrates the distribution of BCS and degree of nostril stenosis against BOAS functional grade.

Fig 5 gives an indication of the effect of each conformational ratio on the probability of BOAS; depicting the univariate logistic curve fit to each ratio individually. In the final model (Table 3), SI (p = 0.016) and NGR (p<0.0001) were significantly associated with BOAS, with odds ratios of 1.05 (95%CI: 1.01 to 1.09) and 1.29 (95%CI: 1.18 to 1.43) for an increase of 0.01 in SI and NGR, respectively.

Fig 6 shows the predictive performance of the final model. The classification was based on a cut-off value of 0.5 (Fig 6A), which showed good accuracy in classifying BOAS (+) and BOAS (-) dogs with AUC of the ROC at 81% (95%CI: 75% to 87%).

In the final model, NGR was retained as a valid predictor in the model (p<0.0001) with good inter-observer agreement of the measurement (ICC = 0.81), as well as a significant predictor for BOAS index (p = 0.001). The mean NGR in male bulldogs (mean = 0.71) was significantly higher than that of female bulldogs (mean = 0.66, t = -7.40, df = 170.9, p<0.0001). The AUC of the ROC for NGR alone was 73% (95%CI: 61–84%) for male bulldogs, which indicates moderate accuracy in classifying BOAS (+) and BOAS (-) dogs. The cut-off NGR value was 0.71 with sensitivity of 71% and a specificity of 69%. NGR was less sensitive in female bulldogs than male bulldogs: the AUC was slightly lower in female at 70% (95%CI: 61–80%). With a cut-off NGR value at 0.66, the sensitivity was 71% and the specificity was lower at 61%.

#### (4) Validation of the model using BOAS index

The BOAS (+) prevalence (functional grade II/III) was not significantly different between all study bulldogs and the bulldogs in the validation group (i.e. the ones that had BOAS index available) (χ^2^ = 1.093, p = 0.296). The results of the multivariate linear regression models can be found in Table 4 (raw data can be found in S3 Data). The adjusted R-squared value of the multivariate linear regression model was 0.26.

Neuter status (p = 0.044), stenotic nostrils (p = 0.003), and NGR (p = 0.001) were significantly associated with BOAS index. Compared to the intact bulldogs, the neutered bulldogs had a mean increase in BOAS index of 24%, although again the 95%CI was wide (1% to 47%). Dogs with moderately/severely stenotic nostrils (compared to open/mild) had a mean increase in BOAS index of 16% (95%CI: 6% to 27%). A 0.01 increase in NGR is associated with a mean increase of 2% (95%CI: 1% to 4%) in BOAS index.

## Discussion

This study describes breed-specific models using several conformational factors to predict the probability of being BOAS-affected. The large sample size in the study supports that each ratio is reliable in population terms, and could guide the writing of breed standards, although the likelihood of inaccuracy in many of the individual measurement as shown by ICC and eME means that most of the individual limits cannot be set. The reliable measures, such as the NGR in bulldogs, and other easily accessible factors, such as nostril stenosis and BCS, may be of use for breeding selection.

### Inter-observer agreement of conformational soft tape measurements

Sutter *et al*. (2008) reported that approximately 0.13% of soft tape measurements used in a multi-breed study were judged to be measurement errors, yet the actual inter-observer agreement of the measurements was not tested. Neither did the Packer *et al*. (2015) study investigating the conformational risk factors for BOAS describe measurement inter-observer agreement. Unfortunately, most of the conformational soft tape measurements in the present study had poor inter-observer agreement in all three breeds. The authors have found that performing tape measurements may be challenging on unsedated dogs, as measurements can be altered easily with slight changes in position (e.g. small changes in degree of the angle between the neck and the back or in head carriage when standing). In dogs with loose and thick skin and/or thick fat coverage it is particularly difficult to reproduce the measurements with good accuracy. Moreover, some of the dogs objected to facial measurements such as SnL. The measurements mentioned above showed large errors of up to 18.7% between two different trained observers and had poor inter-observer agreement according to ICC. These measurement errors directly affect the inter-observer agreement of the respective ratios. The inter-observer reproducibility of the CFR was worst in French bulldogs with the mean measurement errors over 22%. French bulldogs have highly variable over-nose skin fold patterns that affect this measure, whereas in the other two breeds, the type of fold was more uniform (a single fold). Among all the ratios, only CFR in pugs and NGR in bulldogs had reasonably good inter-observer agreement.

### Breed-specific predictors for brachycephalic obstructive airway syndrome

In pugs, female dogs have a higher risk of developing BOAS than male dogs. Interestingly, in French bulldogs, the trend was the opposite. While male dogs are often used as stud dogs at an early age, postponing the decision to breed until the dog is older is recommended, as affected dogs may only show clinical signs in adulthood.

Stenotic nostrils were a significant predictor for BOAS in all three breeds, consistent with our previous findings [16]. Stenosis of the nostrils is the only BOAS airway lesion that can be easily diagnosed without sedation and/or specific equipment, such as an endoscope. The grading system proposed by the authors is straightforward and easily applicable by dog owners. Importantly, nostril stenosis may play a significant role in the severity of BOAS. Nasal breathing is predominant in dogs, even when the dog is panting, the majority of airflow passes through the nasal cavity during inspiration [34, 35]. Commonly, dogs with moderately/severely stenotic nostrils have immobile nostril wings during exercise. Whereas dogs with open/mildly stenotic nostrils usually have mobile nostril wings that can abduct further when needed [15]. Due to the restriction of airflow at the entrance of the airway, dogs with stenotic nostrils can be prone to poor thermal regulation and may have an excessive increase in negative pressure within the airway. Stenotic nostrils are a particular issue in French bulldogs, in the present study, 45% of French bulldogs had severe stenosis of the nostrils. Since the impact of stenotic nostrils on BOAS is substantial, the responsible breeder should avoid using dogs with moderate/severe stenotic nostrils.

Obesity, as quantified using BCS, was a robust risk factor for BOAS, and this result is consistent with previous studies [16, 26]. The impact of obesity on respiratory function includes a decrease in minute volume with an increase in respiratory rate, exercise intolerance, and a decrease in estimated arterial oxygen saturation [16, 36, 37]. Interestingly, while BCS was a significant risk factor for BOAS in pugs and bulldogs, this was not the case in French bulldogs. Only 8.4% of French bulldogs were obese compared to 60.8% and 35.3% of pugs and bulldogs, respectively. About 15% of Grade III French bulldogs were underweight and all of these had frequent regurgitation. In addition to the possible anatomical abnormalities (e.g. oesophageal diverticula), the increase in thoracic negative pressure during respiratory distress could further trigger gastrointestinal signs such as regurgitation and vomiting as a result of gastro-oesophageal reflux and temporary hiatal hernia [38, 39]. Nevertheless, the impact of obesity on BOAS in French bulldogs should not be ignored. It was noted that the majority of the obese French bulldogs were BOAS (+) (Fig 4A).

BOAS, in many ways, is similar to human obstructive sleep apnoea (OSA) [40–43]. The effect of obesity on OSA has been investigated with different measurements. Recently, body mass index (BMI) z-score and neck-to-waist ratio were recognized as independent predictors of OSA [44]. Similar findings were shown in the current study for French bulldogs and bulldogs. Packer *et al*. (2015) have previously reported that a greater neck girth increased the risk of BOAS, but the NG to CG ratio was not significantly associated with BOAS. In the current study, both the absolute measurement of NG and NGR were significantly higher in BOAS (+) French bulldogs and bulldogs (S3 Table). The reason why NG and other direct measurements were not included in the initial models was that the measurements are significantly affected by body size and gender. Although gender was included in all models, the variation in size within the same gender should not be ignored. As the CG was comparable between the BOAS (-) and BOAS (+) dogs, it could be used as a reference to normalize NG. Neck fat accumulation is associated with severity of OSA in humans, and could cause a reduction in pharyngeal lumen diameter, further triggering collapse of the airway [45–49]. Unlike pugs and bulldogs, French bulldogs in our study tended to have an ideal BCS. High NGR in slim dogs could be caused by either fat or muscle. The actual relationship between the NGR and the impact on upper airway obstruction is unknown. Nevertheless, NGR is a strong and valid predictor of BOAS independently of other factors in bulldogs and it may be assumed that selection away from this phenotype will reduce the prevalence of BOAS. In French bulldogs, it was unexpected that the inter-observer agreement of NGR was poor when the measures of NG and CG were both reasonably reproducible. It was found that in some dogs, the observer-1 measured the NG longer but CG shorter than observer-2. Although the differences were not considerable for both of the measurements, the accumulated errors have a significant impact on NGR measurement inter-observer agreement.

Other conformational factors such as SI and EWR in pugs, and NLR in French bulldogs were significantly associated with BOAS in the final models, after adjusting for other factors such as gender. However, not only did these measurements have poor inter-observer agreement, they were also not significantly associated or only marginally associated with the BOAS index. Therefore, the measurements may not be valid for predicting individual BOAS-affected dogs at this stage. However, it is possible, that by introducing more reliable measurement methods these factors may be used as valid predictors for BOAS (+) in the future.

### Craniofacial ratio and brachycephalic obstructive airway syndrome

**Fig 7:**
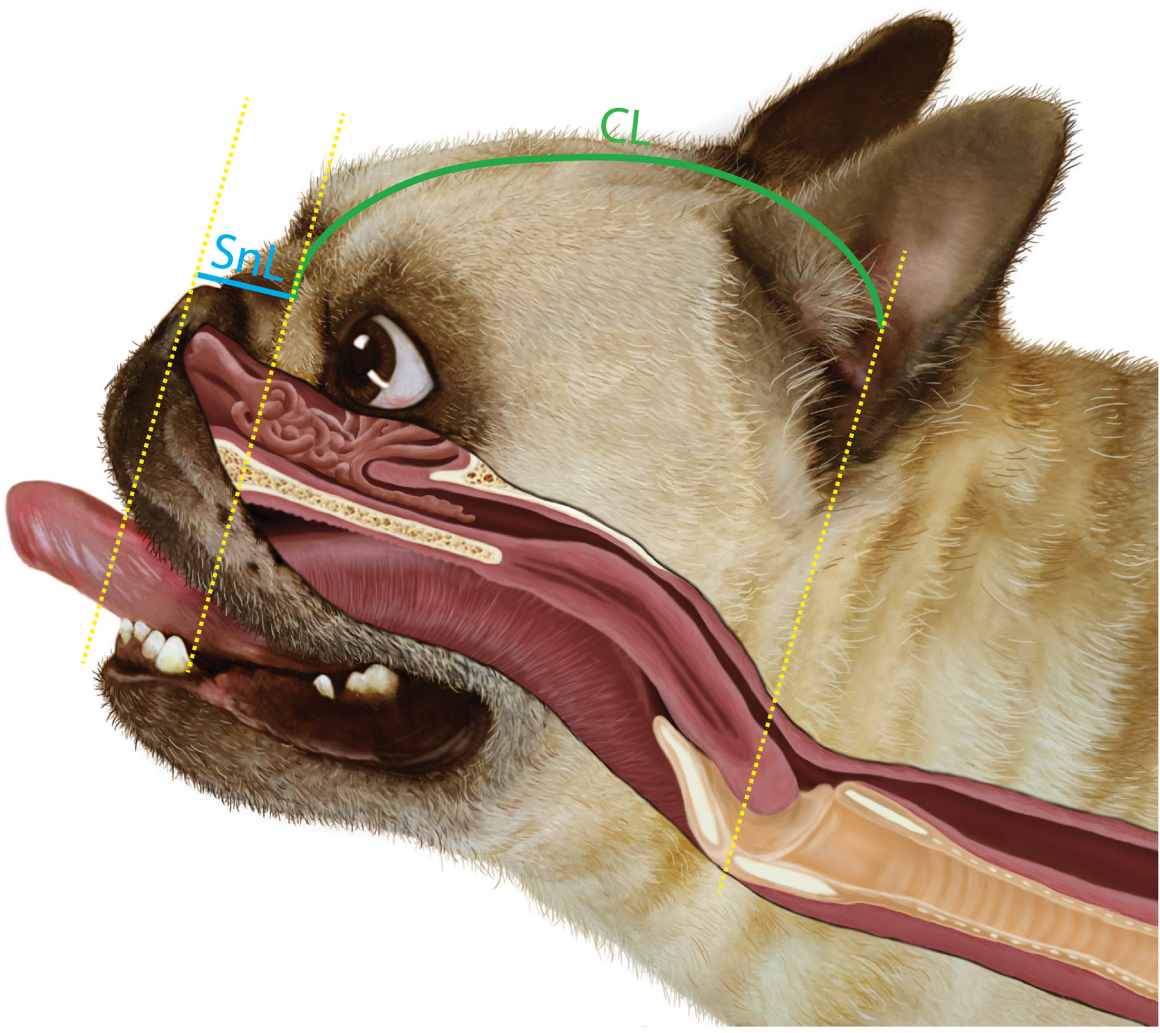
An illustration demonstrates the relationship between the external craniofacial ratio (CFR) measurement and the corresponding internal anatomical structures of the upper airway. The realistic anatomical illustration was made according to a computed tomographic 3-dimensional rendering image of a French bulldog. The illustration was reprinted from the Cambridge BOAS research group website (http://www.vet.cam.ac.uk/boas) under a CC BY license, with permission from the group in the University of Cambridge, original copyright 2016.

A previous study suggested that BOAS risk increases in dogs with relatively shorter muzzles (craniofacial ratio, CFR) and thicker necks, across different brachycephalic breeds: from extreme brachycephalic breeds such as the pug (median CFR = 0.08) to moderate brachycephalic breeds such as the Staffordshire Bull Terrier (median CFR = 0.5) [26]. In our study with large numbers of dogs of the three breeds, we obtained supportive data on NGR, but only a weak association of BOAS status with CFR in a single breed. Within breeds, the variations in CFR were very limited. CFR overlapped considerably between the different BOAS functional grades. Our findings on the reproducibility of these measures and the large differences in detailed conformation between brachycephalic dog breeds suggest that the true associations between CFR and BOAS for specific breeds may not be comparable to the findings in the Packer *et al*. (2015) study that compared multiple breeds with, in most cases, relatively small numbers of dogs. Anatomically, the CFR measurement cannot determine the main internal BOAS lesions along the upper airway. Fig 7 illustrates the position at which CFR measurements are made, in comparison to the position of the internal lesions of BOAS. For extreme brachycephalic dogs, the SnL only includes the region of the nasal planum and nasal vestibule, while other common BOAS lesions such as overcrowded and aberrant nasal turbinates, elongated soft palate, and macroglossia underlie the CL. Therefore it is questionable that having higher CFR would effectively decrease the risk of BOAS for all individuals in the current population. Instead, airway crowding will occur both for individuals with a short facial length and for individuals with a short cranium, so that the most severely affected CFR is not predictable. In dogs that had CFR higher than the third upper-quartile (CFR > 0.19), 46% were still BOAS (+). In contrast to this result, it is more encouraging that among the dogs that had open/mildly stenotic nostrils, only 25% of them were BOAS (+) whereas among the dogs that had moderately/severely stenotic nostrils, 70% of them were BOAS (+). When considering a more effective criterion to assist in breeding away from BOAS, it is likely that the nostril grading would be superior to CFR.

### Limitations of the study

There are several limitations to this study. Firstly, only about half of the study population had an available BOAS index, which markedly reduces the power to validate the final model. Secondly, the study was conducted over three years. It might be possible that the investigators gradually gained more experience on measuring the dogs over time. It should be noted though that the reproducibility measurements were performed towards the end of the study. It is also possible, though unlikely, that the trend of the conformation changed over the study period. Thirdly, as all study dogs were recruited from the UK, the results may have limited significance on these breeds in other regions of the world.

### Conclusion

Nostril stenosis is a strong predictor of BOAS for all three breeds. Dogs with moderate to severe stenosis of the nostrils were at higher risk of developing BOAS. BCS is significantly associated with BOAS in pugs and bulldogs with obese dogs having a higher risk of being BOAS (+). Among the conformation measurements, NGR is a valid predictor of BOAS in male bulldogs and highly reliable between different observers, thus it could potentially be used for breeding selection. EWR and SI in pugs, and NGR and NLR in French bulldogs, SI in bulldogs, were associated with BOAS but had poor-moderate inter-observer reproducibility. Nevertheless, they may be of use for directing the reformation of breed standards.

Overall, the conformational and external factors as measured here contribute less than 50% of the variance that is seen in BOAS. The authors strongly suggest using these in conjunction with regular clinical assessment of respiratory signs before and after exercise (BOAS Functional Grading). More importantly, breeding toward extreme brachycephalic features should be strictly avoided.

## Supporting information

S1 Table The results of the inter-observer mean measurement errors of the conformational soft tape measurements. (DOCX)

S2 Table The results of the inter-observer agreement of the conformational soft tape measurements. (DOCX)

S3 Table Comparison of conformational soft tape measures between BOAS (-) and BOAS (+) dogs. (DOCX)

S1 Fig Boxplots show the distribution of the five conformation ratios against BOAS functional grades. The x-axis is BOAS functional grade; the y-axis is the ratios in percentage. CFR, craniofacial ratio; EWR, eye with ratio; SI, skull index; NGR, neck girth ratio; NLR, neck length ratio.
(TIF)

S1 Data Raw data for the reproducibility tests of soft tape measurements. (XLSX)

S2 Data Raw data for the full model. (XLSX)

S3 Data Raw data for the validation model. (XLSX)

## Abbreviations

AIC: Akaike’s Information Criterion
AUC: area under the curve
BCS: body condition score
BL: body length
BOAS: brachycephalic obstructive airway syndrome
BW: body weight
CFR: craniofacial ratio
CG: chest girth
CI: confidence interval
CL: cranial length
EMMS: electromedical measurement systems
ETT: exercise tolerance test
EW: eye width
EWR: eye width ratio
ICC: intra-class correlation coefficient
MV: minute volume
NG: neck girth
NGR: neck girth ratio
NL: neck length
NLR: neck length ratio
OR: odds ratio
PEF: peak expiratory flow rate
PIF: peak inspiratory flow rate
QDA: quadratic discriminant analysis
ROC: receiver operating characteristic
RR: respiratory rate
SD: standard deviation
SI: skull index
SL: skull length
SnL: snout length
SW: skull width
TBFVL: tidal breathing flow volume loops
Te: expiratory time
Ti: inspiratory time
TV: tidal volume
WBBP: whole-body barometric plethysmography
