# Supplementary material for: Conformational risk factors of brachycephalic obstructive airway syndrome (BOAS) in pugs, French bulldogs, and bulldogs
Source: PLoS One. 2017 Aug 1;12(8):e0181928. doi: 10.1371/journal.pone.0181928 (PMC5538678; doi:10.1371/journal.pone.0181928)
Supplement: S1 Table — (DOCX) [file pone.0181928.s001.docx]

**S1 Table. The results of the inter-observer measurement errors of the conformational soft tape measurements.**

|  | **Pugs (n=20)** | | **French bulldogs (n=20)** | | **Bulldogs (n=20)** | |
| --- | --- | --- | --- | --- | --- | --- |
|  | **Mean (SD)** | **eME,% (SD)** | **Mean (SD)** | **eME,% (SD)** | **Mean (SD)** | **eME,% (SD)** |
| ***Direct measurements (cm)*** | | | | | | |
| **SL** | 11.9 (1.05) | 9.21 (6.91) | 14.61 (1.50) | 8.51 (5.65) | 19.84 (1.0) | 7.52 (5.88) |
| **SnL** | 1.16 (0.40) | 18.56 (19.12) | 2.02 (0.52) | 18.67 (19.43) | 2.38 (0.36) | 16.9 (15.61) |
| **CL** | 10.7 (0.91) | 8.57 (7.79) | 12.59 (1.35) | 9.67 (6.52) | 17.46 (0.9) | 7.08 (5.84) |
| **SW** | 10.81 (1.07) | 7.03 (6.54) | 11.88 (1.45) | 8.03 (6.82) | 14.8 (1.02) | 5.37 (3.87) |
| **EW** | 4.81 (0.48) | 8.53 (6.19) | 5.36 (0.62) | 8.68 (10.76) | 5.8 (0.43) | 8.71 (6.68) |
| **NL** | 10.49 (1.67) | 16.24 (10.25) | 11.15 (1.23) | 12.21 (7.59) | 14.38 (1.95) | 13.33 (10.08) |
| **NG** | 31.91 (2.89) | 6.77 (4.04) | 35.44 (5.66) | 5.69 (5.35) | 51.2 (3.1) | 3.73 (3.31) |
| **CG** | 47.97 (4.60) | 4.75 (3.73) | 51.47 (6.03) | 4.48 (2.58) | 74.92 (3.46) | 2.81 (1.78) |
| **BL** | 31.25 (2.88) | 7.60 (6.10) | 33.33 (6.2) | 7.51 (5.43) | 40.99 (2.68) | 7.41 (3.78) |
| ***Ratios*** | | | | | | |
| **CFR** | 0.11 (0.04) | 18.90 (17.68) | 0.16 (0.04) | 22.06 (19.62) | 0.14 (0.02) | 16.21 (14.18) |
| **EWR** | 0.45 (0.04) | 13.13 (9.75) | 0.46 (0.04) | 14.33 (9.68) | 0.39 (0.03) | 8.81 (7.25) |
| **SI** | 0.91 (0.08) | 10.38 (8.64) | 0.82 (0.1) | 9.70 (7.37) | 0.75 (0.06) | 11.01 (6.39) |
| **NGR** | 0.67 (0.04) | 4.91 (4.58) | 0.69 (0.05) | 6.57 (5.56) | 0.68 (0.05) | 3.68 (3.01) |
| **NLR** | 0.34 (0.06) | 17.60 (15.19) | 0.35 (0.07) | 17.53 (10.1) | 0.35 (0.05) | 15.5 (11.59) |
| SD, standard deviation; SL, skull length; SnL, snout length; CL, cranial length; SW, skull width; EW, eye width; NL, neck length; NG, neck girth; CG, chest girth; BL, body length; ICC, intra-class correlation coefficient; CI, confidence interval.  eME, estimated means of measurement error, calculated from the dataset for the inter-observer reproducibility test. Measurement error (%) = the ratio of measurement difference between the two observations to the mean of the two observations. | | | | | | |
