# Supplementary material for: Conformational risk factors of brachycephalic obstructive airway syndrome (BOAS) in pugs, French bulldogs, and bulldogs
Source: PLoS One. 2017 Aug 1;12(8):e0181928. doi: 10.1371/journal.pone.0181928 (PMC5538678; doi:10.1371/journal.pone.0181928)
Supplement: S2 Table — (DOCX) [file pone.0181928.s002.docx]

**S2 Table. The results of the inter-observer agreement of the conformational soft tape measurements.**

|  | **Pugs (n=20)** | | **French bulldogs (n=20)** | | **Bulldogs (n=20)** | |
| --- | --- | --- | --- | --- | --- | --- |
|  | **ICC** | **95%CI** | **ICC** | **95%CI** | **ICC** | **95%CI** |
| ***Direct measurements*** | | | | | | |
| **SL** | 0.42 | -0.03 to 0.73 | 0.62 | 0.25 to 0.83 | 0.05 | -0.43 to 0.48 |
| **SnL** | 0.83 * | 0.62 to 0.93 | 0.67 | 0.35 to 0.86 | 0.14 | -0.34 to 0.55 |
| **CL** | 0.40 | -0.05 to 0.71 | 0.57 | 0.17 to 0.80 | 0.11 | -0.36 to 0.53 |
| **SW** | 0.63 | 0.27 to 0.83 | 0.70 | 0.37 to 0.87 | 0.62 | 0.24 to 0.83 |
| **EW** | 0.56 | 0.16 to 0.80 | 0.46 | 0.02 to 0.75 | 0.29 | -0.18 to 0.65 |
| **NL** | 0.49 | 0.06 to 0.77 | 0.39 | -0.06 to 0.71 | 0.40 | -0.04 to 0.71 |
| **NG** | 0.68 | 0.36 to 0.86 | 0.89 * | 0.74 to 0.95 | 0.73 | 0.32 to 0.89 |
| **CG** | 0.83 * | 0.62 to 0.93 | 0.91 ** | 0.78 to 0.96 | 0.78 * | 0.37 to 0.92 |
| **BL** | 0.61 | 0.26 to 0.83 | 0.88 * | 0.71 to 0.95 | 0.44 | 0.02 to 0.73 |
| ***Ratios*** | | | | | | |
| **CFR** | 0.84 * | 0.64 to 0.93 | 0.59 | 0.22 to 0.81 | 0.20 | -0.28 to 0.59 |
| **EWR** | 0.19 | -0.29 to 0.58 | 0.08 | -0.40 to 0.50 | 0.41 | -0.04 to 0.72 |
| **SI** | 0.30 | -0.16 to 0.65 | 0.58 | 0.19 to 0.81 | 0.15 | -0.33 to 0.56 |
| **NGR** | 0.44 | 0.02 to 0.73 | 0.41 | 0 to 0.71 | 0.81 * | 0.58 to 0.92 |
| **NLR** | 0.47 | 0.04 to 0.75 | 0.54 | 0.14 to 0.79 | 0.40 | -0.05 to 0.72 |
| SD, standard deviation; SL, skull length; SnL, snout length; CL, cranial length; SW, skull width; EW, eye width; NL, neck length; NG, neck girth; CG, chest girth; BL, body length; ICC, intra-class correlation coefficient; CI, confidence interval.  * Good reliability, an ICC estimate was between 0.75 and 0.9, with lower limit of 95% CI < 0.75  ** Excellent reliability, an ICC estimate was greater than 0.9, with lower limit of 95% CI > 0.75  The lower bounds of the ICC confidence intervals that are below zero, are most likely due to the higher degree of variation within observer, compared to between observer, and the relatively small number of samples in each case. | | | | | | |
