# Supplementary material for: Conformational risk factors of brachycephalic obstructive airway syndrome (BOAS) in pugs, French bulldogs, and bulldogs
Source: PLoS One. 2017 Aug 1;12(8):e0181928. doi: 10.1371/journal.pone.0181928 (PMC5538678; doi:10.1371/journal.pone.0181928)
Supplement: S3 Table — (DOCX) [file pone.0181928.s003.docx]

**S3 Table. Comparison of the conformational soft tape measures between BOAS (-) and BOAS (+) dogs.**

|  | **BOAS (-)** | **BOAS (+)** | **P value** |
| --- | --- | --- | --- |
| ***Pugs*** | | |  |
| **SL (cm)** | 12.91 (1.42) [8.9-17.2] | 12.71 (1.32) [9.7-16.9] | 0.339 |
| **CL (cm)** | 11.87 (1.31) [8-16] | 11.70 (1.27) [9-16] | 0.407 |
| **SnL (cm)** | 1.04 (0.31) [0.4-2] | 1.01 (0.29) [0.4-2] | 0.422 |
| **SW (cm)** | 10.30 (1.2) [7.5-12.5] | 10.41 (1.12) [7-13.5] | 0.524 |
| **EW (cm)** | 4.68 (0.42) [3.5-5.5] | 4.88 (0.56) [3.5 -6.2] | 0.009** |
| **NL (cm)** | 10.31 (1.61) [7.5-14.5] | 9.90 (1.72) [6.5-14.5] | 0.100 |
| **NG (cm)** | 33.40 (3.08) [27-39] | 33.54 (3.33) [27-43] | 0.781 |
| **CG (cm)** | 49.89 (3.87)[40-57] | 50.16 (4.02) [41-62] | 0.657 |
| **BL (cm)** | 32.08 (2.73) [26.5-40.5] | 32.38 (3.03) [26-41] | 0.493 |
| **CFR (%)** | 8.85 (2.67) [2.86-16.67] | 8.69 (2.63) [3.2 – 17] | 0.694 |
| **EWR (%)** | 45.95 (5.69) [35-62.5] | 47.14 (5.67) [34.62-61.11] | 0.177 |
| **SI (%)** | 80.40 (10.79) [56.39-111.11] | 82.60 (10.72) [57.14-105.47] | 0.181 |
| **NGR (%)** | 66.99 (4.18)[56.86-79.17] | 66.96 (5.36) [48.65-93.26] | 0.974 |
| **NLR (%)** | 32.21 (4.68) [22.29-46.77] | 30.81 (6.02) [17-50] | 0.080 |
| ***French bulldogs*** | | | |
| **SL (cm)** | 15.69 (1.60) [12-22.5] | 15.78 (1.44) [12.5-20.5] | 0.658 |
| **CL (cm)** | 13.42 (1.43) [10.5-20] | 13.66 (1.32) [10-17.5] | 0.216 |
| **SnL (cm)** | 2.27 (0.52) [1.2-4] | 2.12 (0.45) [1-3.5] | 0.035* |
| **SW (cm)** | 11.95 (0.87) [10-15.5] | 12.39 (0.93) [10-14.5] | <0.001*** |
| **EW (cm)** | 5.24 (0.56) [4-6.5] | 5.53 (0.65) [4-7.2] | <0.001*** |
| **NL (cm)** | 11.05 (1.55) [8-15.5] | 10.94 (1.67) [6-15.6] | 0.634 |
| **NG (cm)** | 36.66 (3.8) [30-43.5] | 38.66 (3.68) [29-49] | <0.0001*** |
| **CG (cm)** | 54.53 (3.8) [47-66] | 55.09 (4.16) [43-67] | 0.312 |
| **BL (cm)** | 33.68 (4.51) [24.5-46] | 34.89 (4.08) [27-48] | 0.047* |
| **CFR (%)** | 17.04 (4.07) [9.70-33.33] | 15.67 (3.57) [6.67-26.92] | 0.012* |
| **EWR (%)** | 43.99 (5) [33.07-57.14] | 44.78 (5.28) [32.31-59.09] | 0.269 |
| **SI (%)** | 76.78 (8.04) [55.56-100] | 78.91 (6.52) [64.71-104] | 0.042* |
| **NGR (%)** | 67.3 (4.77) [54.76 – 84.66] | 70.26 (5.24) [58.62-85.05] | <0.0001*** |
| **NLR (%)** | 33.31 (6.05) [20-50] | 31.61 (5.14) [18.46-50.85] | 0.034* |
| ***Bulldogs*** | | |  |
| **SL (cm)** | 18.70 (1.88) [15-25] | 18.454 (1.863) [14.5-23.8] | 0.349 |
| **CL (cm)** | 15.70 (1.72) [12-21] | 15.57 (1.74) [12.5-21] | 0.5747 |
| **SnL (cm)** | 3.00 (0.65) [1.5-4.5] | 2.89 (0.66) [1.5-4.2] | 0.238 |
| **SW (cm)** | 14.76 (1.31) [12.5-19] | 15.51 (1.41) [12-19] | <0.001*** |
| **EW (cm)** | 6.26 (0.64) [5-8] | 6.50 (0.76) [5-9] | 0.014* |
| **NL (cm)** | 12.89 (2.39) [8-20] | 12.69 (2.4) [8-18.5] | 0.57 |
| **NG (cm)** | 48.66 (3.81) [38-61.5] | 51.21 (4.28) [39-61] | <0.0001*** |
| **CG (cm)** | 73.66 (4.51) [60-90] | 72.87 (5.54) [49.50] | 0.276 |
| **BL (cm)** | 40.27 (3.97) [32.5-57] | 40.61 (4.82) [32.50-68] | 0.590 |
| **CFR (%)** | 19.31 (4.65) [9.41-30.65] | 18.78 (4.71) [8.78-29.63] | 0.424 |
| **EWR (%)** | 42.64 (5.14) [31.58-53.33] | 42.15 (5.38) [31.07-69.23] | 0.514 |
| **SI (%)** | 79.53 (8.93) [58-103.33] | 84.64 (9.63) [65.12-111.11] | <0.001** |
| **NGR (%)** | 66.13 (4.39) [55.88-81.38] | 70.39 (4.65) [60.76-81.16] | <0.0001*** |
| **NLR (%)** | 32.34 (6.91) [18.18-53.33] | 31.77 (7.4) [14.71-52.94] | 0.5747 |
| Data presented as mean (standard deviation) [minimum – maximum ]  * The conformational value was significantly different between BOAS (-) and BOAS (+) dogs at p<0.05; ** p<0.001; ***p<0.0001 | | |  |
