# Supplementary figures and images for: Conformational risk factors of brachycephalic obstructive airway syndrome (BOAS) in pugs, French bulldogs, and bulldogs
Source: PLoS One. 2017 Aug 1;12(8):e0181928. doi: 10.1371/journal.pone.0181928 (PMC5538678; doi:10.1371/journal.pone.0181928)

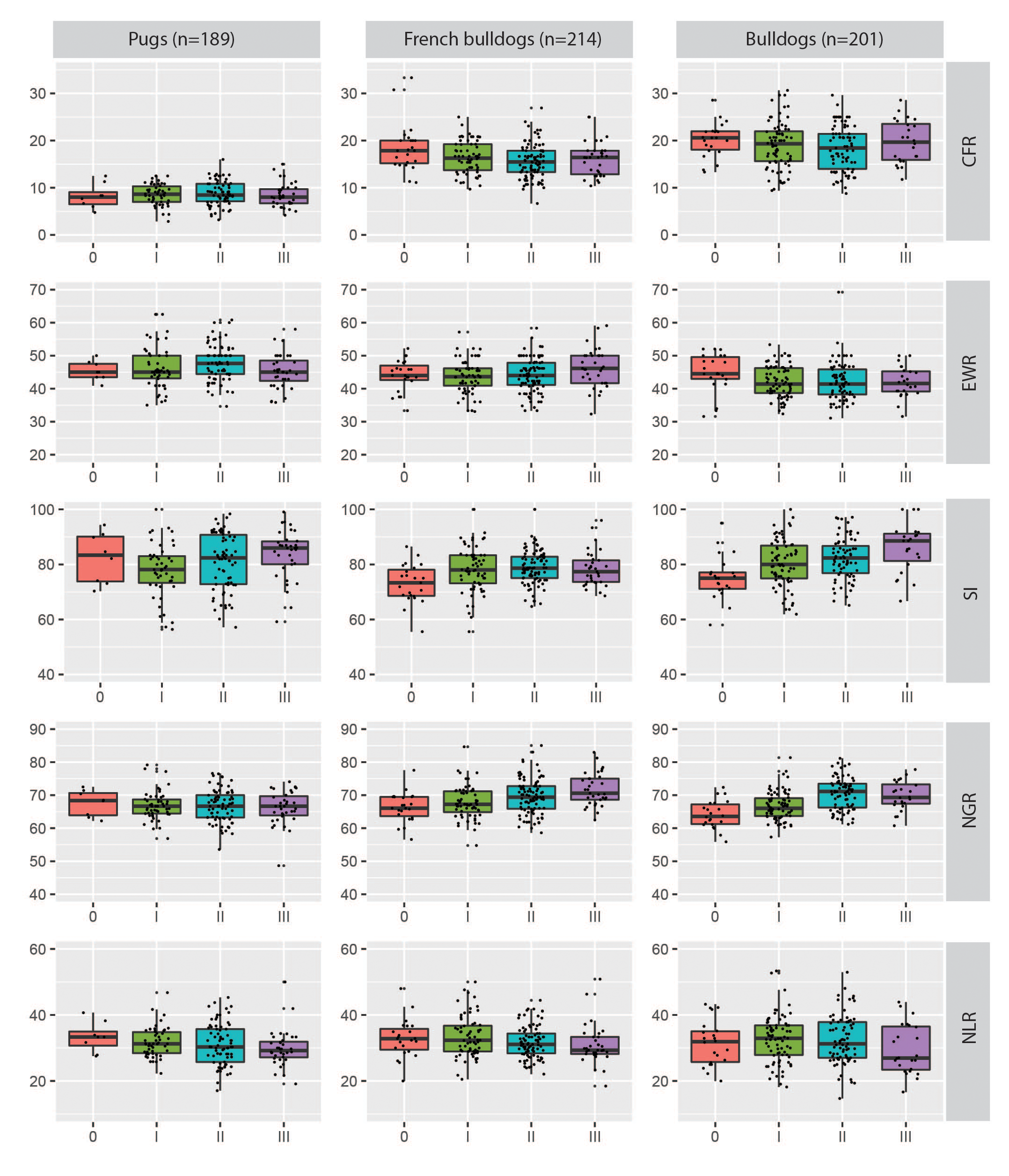

Supplement: S1 Fig — The x-axis is BOAS functional grade; the y-axis is the ratios in percentage. CFR, craniofacial ratio; EWR, eye with ratio; SI, skull index; NGR, neck girth ratio; NLR, neck length ratio. (TIF) [file pone.0181928.s004.tif]
